# Epigenomic landscape of enhancer elements during *Hydra* head organizer formation

**DOI:** 10.1186/s13072-020-00364-6

**Published:** 2020-10-12

**Authors:** Puli Chandramouli Reddy, Akhila Gungi, Suyog Ubhe, Sanjeev Galande

**Affiliations:** Centre of Excellence in Epigenetics, Department of Biology, Indian Institute of Science Education and Research, Dr. Homi Bhabha Road, Pune, 411008 India

**Keywords:** Hydra, Head organizer, Enhancer, Histone modifications, Cis regulatory elements

## Abstract

**Background:**

Axis patterning during development is accompanied by large-scale gene expression changes. These are brought about by changes in the histone modifications leading to dynamic alterations in chromatin architecture. The *cis* regulatory DNA elements also play an important role towards modulating gene expression in a context-dependent manner. *Hydra* belongs to the phylum Cnidaria where the first asymmetry in the body plan was observed and the oral-aboral axis originated. Wnt signaling has been shown to determine the head organizer function in the basal metazoan *Hydra*.

**Results:**

To gain insights into the evolution of *cis* regulatory elements and associated chromatin signatures, we ectopically activated the Wnt signaling pathway in *Hydra* and monitored the genome-wide alterations in key histone modifications. Motif analysis of putative intergenic enhancer elements from *Hydra* revealed the conservation of bilaterian *cis* regulatory elements that play critical roles in development. Differentially regulated enhancer elements were identified upon ectopic activation of Wnt signaling and found to regulate many head organizer specific genes. Enhancer activity of many of the identified *cis* regulatory elements was confirmed by luciferase reporter assay. Quantitative chromatin immunoprecipitation analysis upon activation of Wnt signaling further confirmed the enrichment of H3K27ac on the enhancer elements of *Hv_Wnt5a*, *Hv_Wnt11* and head organizer genes *Hv_Bra1*, *CnGsc* and *Hv_Pitx1*. Additionally, perturbation of the putative H3K27me3 eraser activity using a specific inhibitor affected the ectopic activation of Wnt signaling indicating the importance of the dynamic changes in the H3K27 modifications towards regulation of the genes involved in the head organizer activity.

**Conclusions:**

The activation-associated histone marks H3K4me3, H3K27ac and H3K9ac mark chromatin in a similar manner as seen in bilaterians. We identified intergenic *cis* regulatory elements which harbor sites for key transcription factors involved in developmental processes. Differentially regulated enhancers exhibited motifs for many zinc-finger, T-box and ETS related TFs whose homologs have a head specific expression in *Hydra* and could be a part of the pioneer TF network in the patterning of the head. The ability to differentially modify the H3K27 residue is critical for the patterning of *Hydra* axis revealing a dynamic acetylation/methylation switch to regulate gene expression and chromatin architecture.

## Background

During the development of an organism, the morphology is determined through a series of patterning events discretizing the body via multiple axes such as the anterior/posterior, dorsal/ventral and left/right. The break in the symmetry of the animal Bauplan was the consequence of axis generation. Elegant studies in the past have shown that this is due to molecular polarization via distribution of morphogens into distinct territories creating localized patterning [1, 2]. Highly conserved molecules have been identified as the regulators of the animal body plan which include the *Hox* genes. These homeotic proteins ensure proper patterning and development of an organism and the presence of a cluster of *Hox* genes was thought to be a bilaterian invention [3] The identification and characterization of *Hox* genes in cnidarians and ctenophores suggested that the zootype hypothesis might not be true [4–11]. Further, mechanisms upstream of the *Hox* patterning system were identified and the Wnt/β-catenin signaling pathway was shown to play a critical role in regulating Hox gene expression. Across Metazoa, the Wnt/β-catenin signaling pathway is a part of the posterior patterning during development [12]. Additionally, its function upstream of the *Hox* genes led to postulations that Wnt might be the major player in the primary axis formation [10, 13].

The origin of a defined body axis in the animal kingdom occurred in the phylum Cnidaria that exhibit an oral-aboral axis. Studies in cnidarians and ctenophores have established that unlike in higher phyla, *Hox* genes do not display polarized localization across the axis [10, 14]. In *Hydra* and other cnidarians, the canonical Wnt/β-catenin signaling pathway is known to form the head organizer and recent evidence suggests that it could play an initiating role in the formation of the foot as well [15–18]. This makes *Hydra* an ideal model system to study the process of axis patterning where the primary longitudinal axis is the oral-aboral axis. We have identified the downstream molecular players of the Wnt/β-catenin signaling pathway which resulted in the identification of multiple homeobox transcription factors that play a critical role in *Hydra* axis formation [19].

Regulation of gene expression is orchestrated by multiple short DNA sequences called *cis* regulatory elements that include promoters and enhancers. While promoters are the more proximal regulatory regions and surround the transcription start sites (TSS) of gene bodies, enhancers are distal elements that are located from few Kb to 1 Mb away from the gene body [20]. In genome-wide studies, the combinatorial occupancy of histone modifications is typically used as predictive markers for enhancer identification [21–23]. The typically promoter-associated H3K4me3 modification has also been used as a signature of active enhancers in T-cells [24]. Enhancers can be categorized as active and poised enhancers. Active enhancers predominantly exhibit the H3K4me1 and H3K27ac modifications, whereas poised enhancers possess the H3K4me1 and H3K27me3 modifications [25]. Additionally, acetylation of H3 by p300 and related acetyltransferases on intergenic regions of the genome has been observed at enhancers [26, 27]. Enhancers and promoters can also be discriminated based on the type of RNAs produced from both these regulatory elements. Though both might have bidirectional transcription, enhancers typically produce fewer, shorter and less stable eRNAs restricted to the nucleus whereas promoters usually produce mRNAs that are highly stable, multiexonic, abundant and translated into proteins [28]. Enhancers and promoters also exhibit few sequence-based similarities in terms of transcription factor binding motifs/sites (TFBS). Presence of transcription factor (TF) binding motifs makes enhancers the link between the signals that the cells receive and the target transcription factors that regulate gene expression.

Studying enhancers in the process of axis patterning is critical to understand the mechanisms through which the various signaling processes operate. In *Hydra* the process of axis patterning is also recapitulated during the process of regeneration [19, 29]. During this process, rapid and large-scale alterations in gene expression take place and these require changes in the chromatin architecture regulated by epigenetic modifications and regulatory regions in the genome [30, 31]. Here, we report the conserved characteristics of active histone marks in *Hydra* which have been described previously in bilaterians. We describe the *cis-*regulatory elements that exhibit differential H3K27ac modification in response to systemic activation of Wnt signaling. We also show that the nearest neighboring genes of these DNA elements display head specific expression implying their role in head pattern formation. Further, using a specific pharmacological inhibitor of the JMJD3/UTX enzymes that regulate the demethylation of H3K27me3/2, we demonstrate their role in Wnt signaling mediated phenotypic transformation.

## Results

### Genome-wide occupancy profiles of active histone marks in *Hydra*

**Fig. 1:**
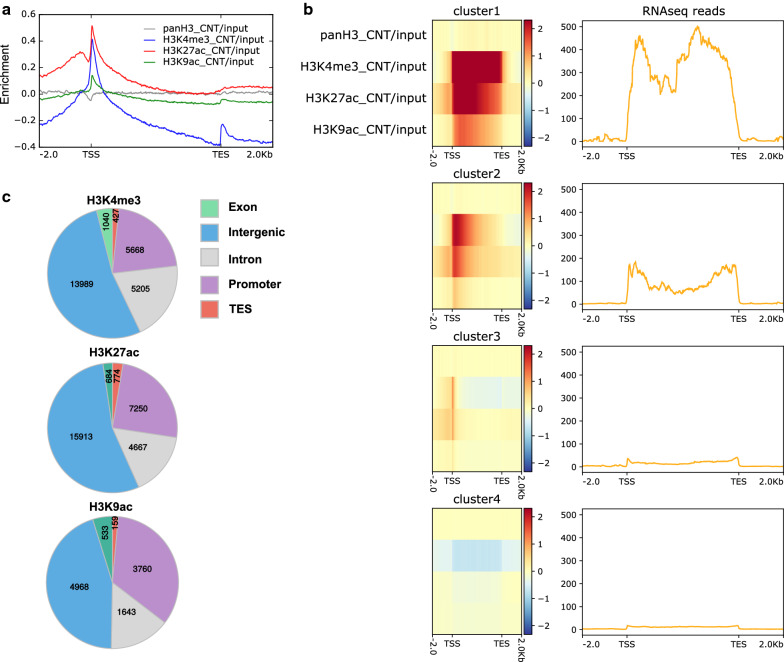
Global histone modifications across gene bodies in *Hydra*. **a** Average profile of the occupancy of the histone H3 and histone modifications H3K4me3, H3K27ac and H3K9ac on genomic regions containing gene bodies (2 Kb upstream and downstream of the transcription start site (TSS) and transcription end site (TES) respectively). **b** Heatmaps representing the four k-means clusters (left) and the average profile of the RNA-Seq reads for the clusters (right). **c** Global occupancy of the three histone modifications H3K4me3, H3K27ac and H3K9ac on different regions of the genome with the numbers representing the number of peaks for the respective histone marks (legend for the colors of the pie-chart is shown on the right)

As a precedent to understanding the role of differential histone modifications in the oral-aboral axis patterning in *Hydra*, it is important to characterize the epigenomic landscape of the head organizer. Previous studies have characterized the histone repertoire in *Hydra* and have identified unique variants such as H2A.X.2 in the gastric region of polyps [32]. In an attempt to understand if there is a unique epigenomic landscape, we sought to characterize the global patterns in histone modifications across the genome of the *Hydra* adult polyps. Towards this goal, we performed chromatin immunoprecipitation followed by high throughout sequencing (ChIP-seq) for activation-associated histone modifications namely, H3K4me3, H3K27ac and H3K9ac. Correlation analysis of the ChIP-seq reads suggests characteristic genomic occupancy specific to each modification (Additional file 1: Fig. S1). We investigated their occupancies at gene bodies, putative promoter regions and intergenic regions. Analysis of the gene bodies in the *Hydra* genome (Fig. 1a and Additional file 2: Table S1) revealed that the occupancy of the activation marks correlates globally with the + 1 nucleosome. Further, depletion of the nucleosome represented by low H3 at the TSS marks the nucleosome-free region (NFR) that allows for accessibility of the promoter regions for binding of RNA Pol II also increases the reliability of this analysis [33]. Based on the combinatorial occupancy of these three histone modifications and reads from the transcriptome analysis using RNA-seq, 4 clusters of genes were identified as seen in Fig. 1b. Cluster 1 is characterized by genes that are highly expressed and therefore have very high levels of H3K4me3 across the transcribing gene body and high levels of H3K27ac and H3K9ac close to the TSS to help recruitment of RNA pol II. The genes in cluster 2 exhibit a very low level of expression and relatively low occupancy of the classical activation marks on histone H3 investigated here. Cluster 3 appears to be genes with no detectable expression but with very low occupancy of both the trimethylation and the acetylation marks on H3. These genes could be the poised genes and will require the knowledge regarding the presence of histone marks associated with repressive functions to establish their identity. The genes in the 4th cluster are characterized by no detectable histone marks and expression, therefore representing the repressed genes. Further, we sought to characterize the intergenic regions of the *Hydra* genome based on the occurrence of activating histone modifications. Upon aligning the ChIP-Seq reads to the *Hydra magnipapillata* genome [34], we obtained the differential occupancy of the three histone marks under investigation across the *Hydra* genome. The genomic distribution of all the three histone modifications revealed the highest number of peaks at the intergenic regions. Additionally, we observed a higher number of intergenic regions with the occurrence of H3K27ac peaks relative to H3K4me3 peaks (Fig. 1c).

### Intergenic regions as enhancers in the *Hydra* genome

**Fig. 2:**
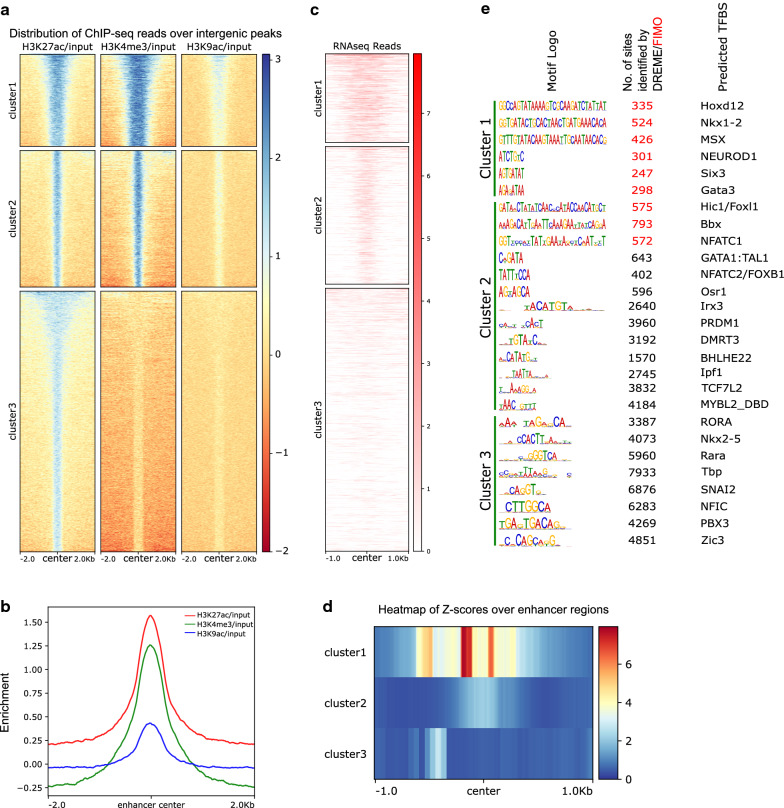
Enhancer identification and TF motif enrichment in the intergenic regions in *Hydra* genome. **a** Heatmap depicting the occupancy of the three histone modifications H3K27ac, H3K4me3 and H3K9ac on the intergenic H3K27ac peaks. Three k-means clusters were obtained based on combinatorial presence of the three modifications. **b** The average profile of the modifications around the center of the peak was plotted for all the intergenic regions. **c** The cluster wise expression of RNA depicted as heatmap based on RNA-Seq reads aligned to the corresponding intergenic regions depicted in ‘**a**’. **d** The Z-scores of the average RNA-seq read profiles for all three clusters over putative enhancer regions. **e** The transcription factor binding motifs/sites (TFBS) that exhibit central enrichment in the intergenic regions of each cluster. The logos of motifs for the TFBS are shown in the first column and the total number of sites identified by DREME (black font) or FIMO (red font) are shown in the second column. The third column shows the transcription factors identified by TOMTOM [35]

Differential occupancy of histone modifications is a part of the regulation of gene expression changes caused by structural changes in chromatin. We retrieved the intergenic regions of the *Hydra* genome excluding the promoters, which displayed the occurrence of H3K27ac as a typical marker for enhancers and investigated the distribution of the three histone modifications namely H3K4me3, H3K27ac and H3K9ac on these intergenic regions. We plotted the occupancy of the three histone modifications on all the intergenic regions and obtained three k-means clusters based on the distribution pattern of the modifications around the peak center (Fig. 2a and Additional file 3: Table S2, Additional file 4: Table S3). Cluster 1 consists of 2884 intergenic regions with a higher level of H3K4me3 than H3K27ac and the distribution of the marks occurs in a broad peak. Cluster 2 consists of 4475 intergenic regions which also exhibits a higher level of H3K4me3 than H3K27ac, however, the distribution of the marks is in sharper peaks. The 8854 regions in cluster 3 have an epigenetic signature where the level of H3K27ac is higher and H3K4me3 is absent. We then plotted the intergenic regions and the average distribution of the histone modifications centering the peaks for each histone mark (Fig. 2b and Additional file 3: Table S2). To determine the presence of transcription reads from the intergenic regions, we analyzed the RNA-seq reads originating from these regions. The RNA-seq reads in Fig. 2c depict transcription arising from the corresponding intergenic regions in Fig. 2a and the 3 clusters shown are representative of the k-means clusters generated based on the distribution of the histone modification. We observed very high levels of transcription arising from the intergenic regions of cluster 1 (Fig. 2c) in addition to the wide peaks of H3K27ac occupancy (Fig. 2a). Additionally, the occurrence of transcripts from multiple locations across the intergenic DNA elements was also observed as seen in the heat map provided (Fig. 2d). The second cluster obtained also displayed transcription of RNA which was restricted usually to a single originating location and was not spread across the DNA element (Fig. 2c, d). This pattern was similar to the pattern of occupancy of the histone marks on these intergenic regions (Fig. 2a). The third cluster did not show any transcriptional activity and the lack of H3K4me3 mark was striking in this case with a presence of the H3K27ac mark (Fig. 2a, d) which is seen in the regions in cluster 3 of Fig. 2a, c. To understand the role of these intergenic regions in transcriptional regulation, we identified the TFBS motifs present in these regions using the MEME-ChIP tool in the MEME Suite package. The motifs across the three clusters which exhibit central enrichment of TFBS in a 500 bp DNA element around the histone modification peak are represented (Fig. 2e, Additional file 1: Fig. S2). Multiple TFBS were identified on the intergenic regions and most of these have a developmental role like the homeodomain-containing TFs, Nkx1-2, Irx3 and PBX3 [93–95]. Notably, we observed significant enrichment of TCF7L2, which is a key target transcription factor of the head patterning Wnt/β-catenin signaling pathway in *Hydra,* on multiple intergenic regions in the 2nd cluster (Fig. 2e, Additional file 1: Fig. S2).

### Intergenic genomic DNA is differentially modified for the process of axis patterning in *Hydra*

**Fig. 3:**
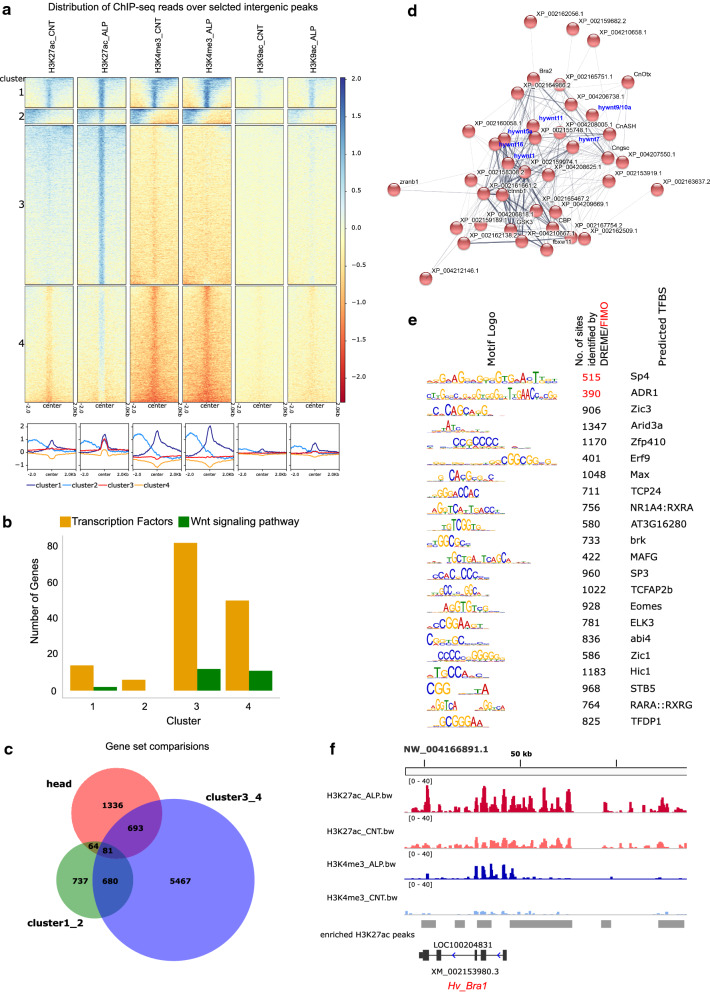
Dynamic regulation of enhancer-like elements during axis patterning in *Hydra*. **a** The occupancy of the H3K27ac, H3K4me3 and H3K9ac on the enriched peak regions identified by activation of Wnt signaling using Alsterpaullone (ALP). Four k-means clusters were obtained based on the combinatorial presence of the three modifications. The average profile of the modifications is depicted below the heatmap of each modification with the colors denoting different clusters. **b** Distribution of Gene ontology (GO) terms for transcription factors (GO:0003700, GO:0043565) and Wnt signaling pathway members (GO:0016055) for the nearest neighbour genes of the intergenic regions across the four clusters. **c** Comparison of head specific genes identified by performing differential gene expression analysis using RNA-Seq from head and foot regions (SRR6815024-29) with the nearest neighbour genes of the intergenic regions from clusters 1 & 2 (cluster1_2) and clusters 3 & 4 (cluster3_4). **d** The 774 head specific genes which exhibit enriched H3K27ac mark upon ALP treatment were subjected to network analysis using STRING [38]. Cluster analysis of the network generated showed a large cluster enriched with Wnt signaling pathway members. **e** The transcription factor binding motifs/sites (TFBS) that show central enrichment in the intergenic H3K27ac peak regions of the 774 genes (intersection of head specific and cluster3_4 genes). The logos of motifs for the TFBS are shown in the first column and the total number of sites identified by DREME (black font) or FIMO (red font) are shown in the second column. The third column shows the transcription factors identified by TOMTOM. **f** Integrative Genomics Viewer (IGV) [39] track plots depict differential occupancy of H3K27ac and H3K4me3 upon ALP treatment on the gene body and upstream regulatory regions of *Hv_Bra1* which plays a role in head patterning in *Hydra*. The regions showing enriched H3K27ac peaks are depicted in grey in the last track

The oral-aboral axis of *Hydra* is characterized by the molecular and morphological distinction of the body column into the head and foot structures. The canonical Wnt/β-catenin signaling pathway plays a critical role in the formation of the head organizer and differentiation of the associated structures such as tentacles and mouth. To understand the molecular mechanisms underlying the head formation and axis patterning in *Hydra*, we used Alsterpaullone (ALP), a GSK3β inhibitor to induce systemic activation of the Wnt/β-catenin signaling pathway in the polyps. Following activation of the Wnt pathway, to decipher the epigenetic regulation of transcription, we performed ChIP-seq for H3K4me3, H3K27ac and H3K9ac in both treated and control animals. We then retrieved intergenic regions which show increased occupancy of H3K27ac upon activation of Wnt signaling (Additional file 5: Table S4), which is a classical enhancer associated mark, and monitored the occupancy of H3K4me3 and H3K9ac on these intergenic regions. Based on the differential occupancy of the three marks on the intergenic regions, we obtained four clusters of DNA elements using k-means based clustering (Fig. 3a, Additional file 5: Table S4). Depicted below the heatmap in Fig. 3a is the average profile of the occurrence of the histone modification in each cluster. The intergenic regions in the clusters 1 and 2, containing 1220 and 601 sequences respectively, display high levels of H3K27ac, H3K4me3 and H3K9ac marks distributed in relatively broad regions surrounding the peak center. There are 6459 intergenic regions belonging to cluster 3 which show occupancy of H3K27ac in sharp peaks and moderate levels of H3K9ac and this cluster shows clear increase in the levels of H3K27ac and H3K9ac upon the ectopic activation of Wnt signaling pathway. The level of H3K4me3 also increases but much more subtly (Fig. 3a). Cluster 4 contains 4729 elements that show an interesting pattern of histone modifications on the intergenic regions. H3K27ac on these intergenic regions is gained upon ALP treatment and H3K9ac levels show a slight reduction in their levels. These regions are depleted of H3K4me3 occupancy following ALP treatment (Fig. 3a). To understand the role these intergenic regions play in transcriptional regulation during axis patterning, we retrieved the genes located nearest to these regions in all 4 clusters. Gene Ontology (GO) analysis enabled the identification of number of transcription factors and Wnt signaling pathway members in all the clusters (Fig. 3b, Additional file 6: Table S5). The clusters 3 and 4 show greater numbers of transcription factors and Wnt signaling pathway members with dynamic regulation via *cis-*regulatory DNA elements. We compared the genes closest to the intergenic regions in the combined cluster1_2 (clusters 1 & 2) and the combined cluster3_4 (clusters 3 & 4) with head specific genes obtained by comparing publicly available RNA-Seq datasets of *Hydra* head and foot. Here we observed that there are 774 genes that are common between the head and cluster3_4 which correlates with the presence of a larger number of Wnt signaling pathway genes important for head patterning (Fig. 3c, Additional file 1: Fig. S3, Additional file 7: Table S6). Using these 774 genes, a network and clustering analysis was carried out for 237 available orthologs using the STRING database. Following a network enrichment analysis for KEGG pathways, an enrichment of the Wnt and Notch signaling pathway members was observed (Fig. 3d, Additional file 1: Fig. S4, Additional file 8: Table S7). The Wnt signaling pathway was identified as the largest cluster with highly significant interactions and as seen in the network, loci encoding multiple *wnt* ligands were shown to exhibit differential H3K27ac occupancy on their neighboring intergenic regions. Along with the ligands, neighboring intergenic regions of loci encoding multiple transcription factors such as *CnGsc*, *HyBra1*, *CnASH and CnOtx* also display similar differential histone modifications. We performed a motif enrichment analysis using MEME-ChIP on the 774 regions that are common to the head and clusters 3 and 4 and the motifs that show a central enrichment flanking the peak center have been represented in Fig. 3e (Additional file 1: Fig. S5). A majority of the TFs that have been identified belong to the Zinc-coordinating DNA-binding domains superclass of TFs and many of the TFs also have roles in axis patterning, matrix remodeling and nervous system development. Further, multiple TFs that interact with components of the Wnt signaling pathway such as Hic1 and Elk3 were found bind to many intergenic regions [77]. We then investigated the dynamic nature of the histone modifications on few genes that are critical in establishing and maintaining the head organizer activity. *Brachyury* is a T-box transcription factor that plays a role in tissue patterning during budding and head regeneration in *Hydra* [36, 37]. Upon constitutive activation of Wnt signaling, *Brachyury* is upregulated and shows an increase in the occurrence of the histone modifications across the gene body with a greater distribution of the histone modifications proximal to the promoter (Fig. 3f).

### Identified *cis* regulatory elements exhibit enhancer activity

**Fig. 4:**
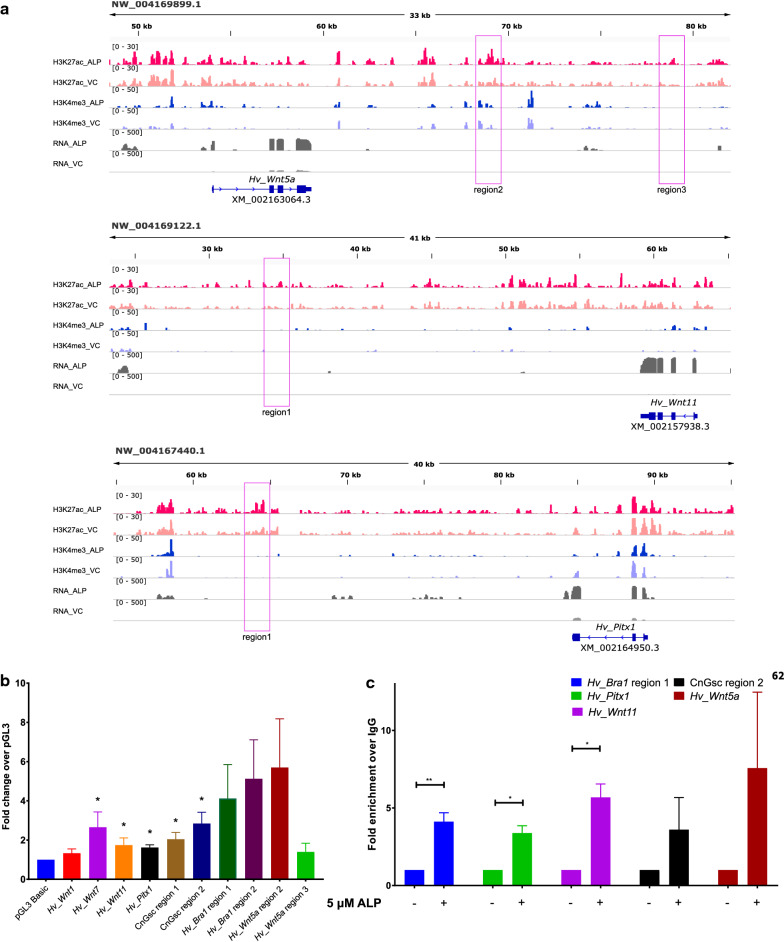
Enhancer activity of *cis*-regulatory elements upstream to multiple Wnts and head organizer genes. Putative enhancer elements were identified based on differential modification of H3K27ac after systemic activation of Wnt signaling and validated by luciferase assay. **a** Plots of the genomic regions generated using IGV depicting occupancy of the H3K27ac, H3K4me3 histone modifications and RNA expression on distal genomic regions of *Hv_Wnt11*, *Hv_Wnt5a* and *Hv_Pitx1*. ALP-Alsterpaullone treated and VC-vehicle control. The magenta rectangles show the regions cloned into the reporter construct for performing luciferase reporter assays and quantitative ChIP-PCRs. **b** The relative luminescence for each of the genomic regions is plotted using pGL3-basic as the control (first column) (** indicates a *p* value < 0.01 using Student’s t-test). **c** Relative enrichment of the occupancy of H3K27ac on the neighboring intergenic regions for the target regions has been plotted as fold enrichment over the IgG control in both DMSO controls (− 5 µM ALP) and ALP treated polyps (+ 5 µM ALP) (* indicates a *p* value < 0.05 and ** indicates a *p* value < 0.01 using Student’s t-test)

Next, we selected genes that play a critical role head organizer activity with putative enhancer activity and monitored the histone modification profiles of their upstream genomic regions (Fig. 4a). In vitro luciferase assay was performed in HEK293T cells using upstream intergenic (not promoter) regions identified based on H3K27ac modification for genes that are a part of the Wnt signaling network. The upstream genomic regions of *Hv_Wnt5a*, *Hv_Wnt7*, *Hv_Bra1* and *CnGsc* which are expressed near the head organizer or tentacles in *Hydra* exhibited significant enhancer activity (Fig. 4b). Additionally, to validate the enrichment of H3K27ac over these regions in response to systemic activation of Wnt signaling we performed quantitative ChIP-PCR analysis. Here, all the regions could not be tested successfully due to failed amplification during PCR. This could be due to repeated sequences observed in this region. However, except *Hv_Wnt7*, the other Wnts (*Hv_Wnt5a* and *Hv_Wnt11*), the head organizer gene *Hv_Bra1* and *Hv_Pitx1* exhibit enrichment of H3K27ac (Fig. 4c). This confirms the regulation of these genes by enhancer activity associated with the identified *cis-*regulatory elements. The differences observed in the luciferase assay and ChIP-PCRs could be due to the variations in the regulatory mechanisms involved under in vivo conditions.

### Differential modification of H3K27 regulates axis patterning in *Hydra*

**Fig. 5:**
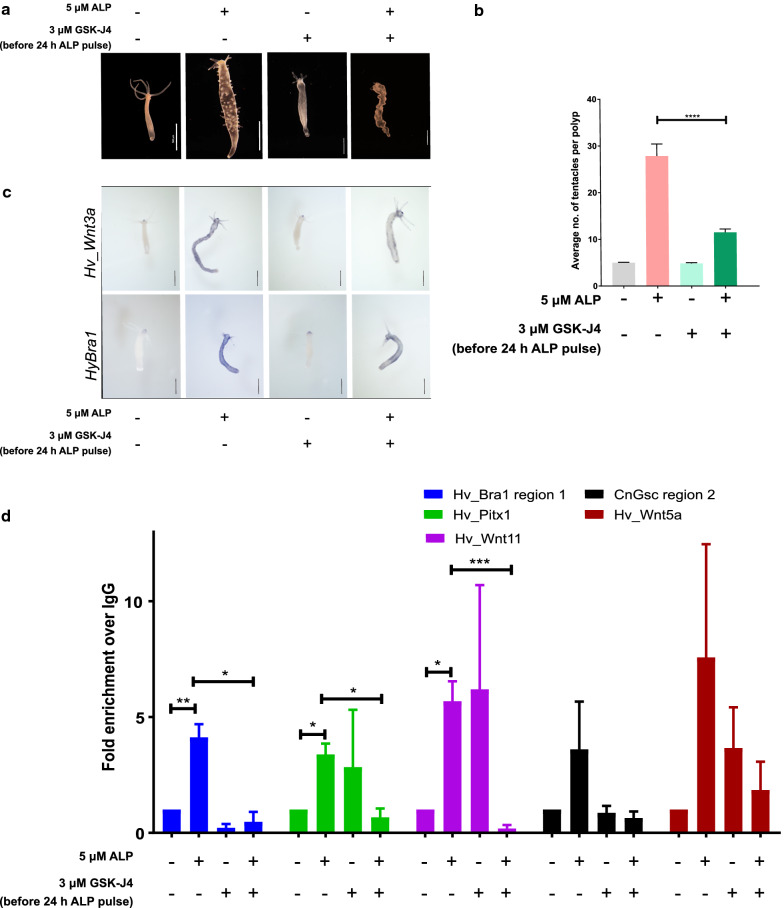
Effect of inhibition of the histone demethylase activity of KDM6A/6B on axis patterning in *Hydra.* **a**, **b** Effect of the pharmacological inhibitor on the ectopic tentacle phenotype which is resulted by ALP treatment. Treatment with the inhibitor of KDM6A/6B (3 µM GSK-J4) results in a significant decrease in the number of tentacles obtained due to ALP treatment. Scale bar = 500 µm. **** indicates a *p* value < 0.0001 estimated by a one-way ANOVA test. **c** Whole mount in situ hybridization for *Hv_Wnt3a* and *HyBra1* shows decreased ectopic organizer centers (blue spots) formed when polyps are treated with GSK-J4 in combination with ALP. Scale bar = 500 µm. **d** Relative enrichment of H3K27ac occupancy on the neighboring genomic regions of target genes with enhanced occupancy upon ALP treatment. Upon combinatorial treatment with GSK-J4 and ALP, the level of H3K27ac shows reduced occupancy on the neighboring intergenic regions. * indicates a *p* value < 0.05, ** indicates a *p* value < 0.01 and *** indicates a *p* value < 0.001 using Student’s t-test (the first two bars have been reproduced from Fig. 4c to indicate changes in H3K27ac occupancy observed upon ALP treatment alone)

In *Hydra*, the canonical Wnt/β-catenin signaling pathway acts as the head organizer and ectopic activation of this pathway is achieved by inhibiting the GSK-3β kinase using Alsterpaullone (ALP). ALP treatment results in multiple molecular and structural changes in the polyp leading to the transformation of the entire body column into a head by giving rise to mis-localized tentacles [40]. As has been previously discussed, such molecular changes involve large-scale global changes in the epigenomic landscape in the cells. To understand the role of specific histone modifications in the axis patterning in *Hydra*, we used a specific pharmacological inhibitor for KDM6A/6B and activated the Wnt/β-catenin signaling pathway using ALP. We observed that when the ALP treated polyps were subjected to the specific inhibitor GSK-J4 that acts against the histone lysine demethylases (KDMs) UTX/JMJD3 (KDM6A/KDM6B) [41], it resulted in a significant reduction in the formation of ectopic tentacles all over the body column of the polyps (Fig. 5a, b, Additional file 1: Fig. S6). Histone modifications have been shown to occur in a stepwise manner and removal of the trimethylation at H3K27 is essential for the acetylation to occur at the same residue. KDM6 demethylases are implicated in the demethylation of the repressive H3K27me3 mark that enables deposition of the H3K27ac mark which results in the activation of repressed genes [42, 43].

## Discussion

The post-genomic era evidenced a shift in focus to non-coding regulatory sequences (*cis* regulatory elements—CREs) primarily due to their paramount contribution towards the differential regulation of gene regulatory networks (GRNs) comprising conserved gene repertoires in an organism/cell type-specific manner. It is fairly established that CREs play a critical role in the evolution of morphological and cell type diversity [44–47]. Among CREs, enhancer elements in combination with promoter elements control gene expression pattern in spatiotemporal fashion and act as substrates for the evolution of novelties such as morphological and functional diversities [48, 49]. Most of the studies related to enhancers are limited to selected model organisms. Owing to this, their divergence at the base of metazoans, where major evolutionary transitions took place with respect to developmental processes such as axis pattering and cell type functions, is poorly understood. Until now very few studies have reported the identification of the enhancer elements from the basal metazoan phylum Cnidaria suggesting their roles in gene regulation in different paradigms [31, 50, 51].

Here, we have characterized the histone modification profiles for three histone marks—H3K27ac, H3K9ac and H3K4me3 typically associated with transcriptional activation [52, 53] in *Hydra*, a member of the phylum Cnidaria. The level and distribution of the histone modifications across the gene body indicates the status of transcription and is different for genes depending on their function [54–56]. Based on the occupancy of the activation-associated histone marks, four clusters of genes have emerged among which the first two represent the high and intermediate level of expression and a fourth cluster representing the repressed genes. The third cluster presumably depicts a set of poised genes that might be important for the perpetual developmental processes occurring in the *Hydra* body column [57]. These genes can be activated or repressed based on signaling stimuli for specific developmental and regenerative processes in a context-dependent manner in *Hydra*. The occupancy of these histone modifications on a large fraction of the intergenic regions in the genome indicates putative enhancer elements in the *Hydra* genome. Enhancers are a critical part of the *cis* regulatory elements for coordinated regulation of gene expression in a signal-dependent and cell-specific manner. Enhancers have been identified and shown to have a conserved mode of function in *Nematostella*, another Cnidarian [58]. Based on the occupancy of the classical enhancer associated histone mark, H3K27ac, we identified three types of enhancer-like intergenic regions in the *Hydra* genome. The first cluster exhibits features of super enhancers with broad peaks of H3K27ac, H3K4me3 occupancy and high levels of transcription with multiple originating locations. The second cluster shows the characteristics of typical enhancers with sharp peaks of the activating histone marks and enhancer RNA like transcription events. The third cluster is unique in that it displayed high H3K27ac marks, but no transcription-associated H3K4me3 marks or transcription for the DNA elements. This has been observed in some super enhancers which respond to specific signaling stimuli and do not have an enhancer RNA transcription [59]. We have also identified multiple TFBS on the intergenic regions that belong to TFs involved in development and programming processes in organisms. The TCF7L2 binding site was also identified in the cluster which is the target TF of the head patterning signaling network in *Hydra* and responds to the Wnt/β-catenin signaling stimulus [19].

Upon activation of the canonical Wnt/β-catenin signaling pathway in *Hydra*, we observed differential histone modification on multiple intergenic regions. We identified four clusters based on the differential active histone modifications. Multiple transcription factors and Wnt signaling pathway genes were identified close to the intergenic regions showing changes in their modification status. Among the Wnt signaling pathway members are multiple *wnt* ligands and many critical transcription factors that play a role in the patterning processes as a response to Wnt signaling [60–65]. The TFBS showing enrichment on the “head-related” intergenic regions have a majority of Zinc finger family of transcription factors which have been known to play roles in patterning processes. Multiple Zic proteins were identified to have TFBS on the Wnt responsive intergenic regions which can bind to multiple transcription factors that play a role in developmental signaling [66–70], chromatin remodeling proteins [71, 72] and many nuclear factors. Additionally, the consensus binding motifs for Zic family TFs have been shown to be enriched in enhancers and involves binding to chromatin remodelers [71, 73–76]. Further, we have identified the TF like *brk* that has a role in transcriptional regulation and the TFs Hic1 and Elk3 that interact with the Wnt signaling pathway to have TFBS in the differentially modified intergenic regions [77].

We have established the enhancer activity for four genomic regions upstream of genes that play a critical role in head patterning namely *Hv_Wnt7*, *Hv_Wnt5a*, *CnGsc* and *Hv_Bra1*. Previous studies have shown that the *Hv_Wnt11*, *Hv_Wnt7*, *Hv_Bra1* and *CnGsc* are expressed in the *Hydra* head organizer [78]. *Hv_Wnt5a* is localized to epithelial cells which give rise to tentacles during bud development [79] and *Hv_Pitx* is localized to and important for the organizer formation in the budding zone [80]. This indicates the spatiotemporal regulation of chromatin via H3K27ac and the regulation of these genes during the process of axis determination in an enhancer-mediated manner. To achieve this type of regulation these genes need to be in relatively close proximity in the genome. However, none of these genes could be located in the same scaffold. This could be presumably due to highly fragmented assemblies of current *Hydra* genomes available [34]. Chromosome level assembly of *Hydra* genome will greatly facilitate the studies on *cis-*regulatory elements.

**Fig. 6:**
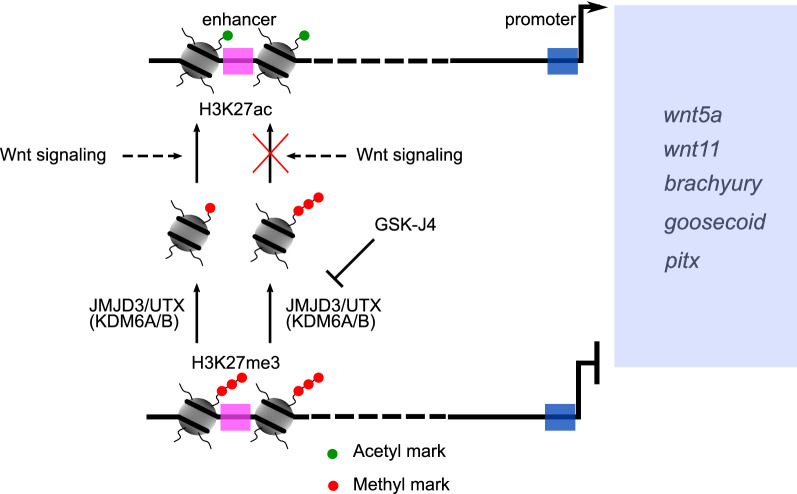
Graphical summary showing the H3K27ac mediated activation of *Hydra* homologs of *wnt5a*, *wnt11*, *brachyury*, *goosecoid* and *pitx*. Wnt signaling causes enrichment of H3K27ac on the enhancer-like elements and it is associated with transcriptional activation. Upon inhibition of the histone demethylase JMJD3/UTX (KDM6A/B), the intergenic *cis-*regulatory regions cannot be demethylated and are therefore not accessible for deposition of the acetyl mark on the H3K27 residue. This leads to the downregulation of the Wnt target genes and thereby disrupts Wnt directed morphogenesis in *Hydra*

Further, we have used the H3K27 methylation mark as a paradigm to assess the differential modification of H3K27ac. The change of post-translational modifications on histones from acetylation to methylation has been implicated in regulating gene expression for the processes of epithelial-mesenchymal transition and differentiation in cancers and neuronal progenitors respectively [81, 82]. We observed genome-wide changes in the H3K27 acetylation status in *Hydra* upon activation of the Wnt/β-catenin signaling pathway which resulted in both the ectopic tentacle phenotype and an expansion of the expression domain of the Wnt target genes like *Hv_Wnt3a* and *Hv_Bra1*. Disruption of the histone demethylation by pharmacological inhibition of JMJD3/UTX (KDM6A/B) leads to a rescue of the ectopic tentacle phenotype caused by the systemic activation of the Wnt/β-catenin signaling. This is a result of an altered ability of the H3K27 residue to be acetylated due to disruption in its ability to be demethylated, which transmutes the manifestation of the molecular and morphological changes in *Hydra*. We propose this H3K27 ac/me switch between the acetylated (ac) and the methylated (me) states as a critical player in the epigenetic regulation of the Wnt/β-catenin mediated axis patterning in *Hydra* (Fig. 6).

## Conclusion

In summary, we have identified the intergenic regulatory elements that possess enhancer-like histone modification signatures such as H3K27Ac and specifically characterized the enhancers that are responsive to Wnt signaling. These putative enhancers are enriched in binding motifs for multiple TFs, among which a majority that belong to the Zinc coordinating domain-containing superfamily with that have been shown to play important roles in developmental processes. Additionally, many TFs known to associate with components of the Wnt signaling pathway such as Hic1, T-box and Ets members have binding motifs on the differentially modified intergenic DNA elements. This investigation also resulted in the identification of candidate pioneer transcription factors that regulate gene expression via *cis-*regulatory elements in response to Wnt signaling in *Hydra*. We have demonstrated the in vitro enhancer activity of the *cis*-regulatory elements for *Hv_Wnt7*, *Hv_Wnt5a*, *CnGsc* and *Hv_Bra1.* Further, we suggest a role of covalent modification of the histone residue H3K27 as a switch to gain acetylation or methylation and facilitate regulation of the Wnt responsive genes in a spatio-temporal manner.

## Methods

### *Hydra* culture

A clonal culture of *Hydra vulgaris* Ind-Pune was maintained at 18 °C using standard protocols described previously [83, 84]. *Hydra* polyps were fed with freshly hatched *Artemia nauplii* larvae daily and cleaned 6–8 h post feeding.

### Alsterpaullone treatment

*Hydra* polyps were starved for 24 h and subjected to 5 μM Alsterpaullone (ALP) (Sigma–A4847) in hydra medium for 24 h. Control treatments with hydra medium and DMSO as a vehicle control were performed. Alsterpaullone was removed after the pulse and this time point is considered as 0 h post treatment. The polyps were incubated in hydra medium for an additional 72 h to observe the morphology of the polyps.

### Inhibitor treatments

Fifty polyps were used for each of the inhibitor treatments. The chemical inhibitor GSK-J4 (for KDM6A and KDM6B) was obtained from Sigma Aldrich (Cat. No. SML0701). GSK-J4 was used at a final concentration of 3 µM to perform all the experiments. The polyps were treated with the demethylase inhibitor for 24 h before the ALP treatmnetw as started. The demethylase inhibitor was changed every day and the treatment continued for 96 h while the ALP treatment was performed as previously mentioned. After 96 h, the animals were fixed in 4% PFA overnight at 4 °C following relaxation with 2% urethane. The polyps were stored in 50% glycerol and imaged. For whole mount in situ hybridization, treatment with GSK-J4 was performed 24 h before the ALP pulse and the polyps were fixed 24 h after the ALP pulse.

### Chromatin immunoprecipitation (ChIP)

Two thousand *Hydra* polyps each for DMSO control and ALP treatment were cross-linked with 1% formaldehyde, lysed and sonicated in sonication buffer (10 mM Tris–HCl pH 7.5, 200 mM NaCl, 1% SDS, 4% NP-40, 1 mM PMSF) to obtain an average chromatin size of 300 bp. Chromatin was pre-cleared using 50 μl of a 50% protein A sepharose (GE healthcare) slurry for 1 h at 4 °C with gentle inverting. Immunoprecipitations were carried out in 1 ml of ChIP buffer (20 mM Tris–HCl pH 8.0, 150 mM NaCl, 2 mM EDTA, 1% Triton-X 100) with Anti-H3K4me3, Anti-H3K27ac, Anti-H3K9ac and Anti-H3. An appropriate IgG control was also used with inverting at 4 °C for 14–16 h. The samples were then incubated with 50 μl of a 50% Protein A sepharose slurry (saturated with 0.5% BSA and 10 mg/ml yeast tRNA) for 3 h at 4 °C with gentle inverting. ChIP samples were reverse-crosslinked, and the DNA was purified using a Qiaquick column (Qiagen). Input chromatin was obtained after preclearing, by de-crosslinking and purifying input DNA using a Qiaquick column (Qiagen) according to manufacturer’s instructions. Purified DNA was subjected to library preparation for sequencing or used for quantitative PCR. The fold enrichment was calculated using the formula—Fold enrichment = 2^(Ct (Target antibody)−Ct (IgG))^. The details of the primers used to perform the ChIP RT-PCR are provided in Additional file 9: Table S8.

### ChIP sequencing

ChIP-seq was performed using the SOLiD 5500 platform (Applied Biosystems). ChIP-seq libraries were prepared from 5 ng of DNA extracted after chromatin immunoprecipitation for different histone modifications using fragment library construction kit from Life technologies. Here, end repairing of the ChIPed DNA was carried out and then adaptors were ligated using T4 DNA ligase. Adaptor ligated products were amplified using adaptor specific primers for 12 cycles. As per the manufacturer’s protocol DNA purification was performed using Agencourt XP beads (Beckman Coulter). Quality and quantity of the libraries were analyzed using Agilent bioanalyzer 2100 high-sensitivity DNA kit. The pooled libraries were used for sequencing.

### ChIP-seq analysis

*Hydra vulgaris* reference genome from NCBI was downloaded (GCF_000004095.1_Hydra_RP_1.0) and used for alignment and other analysis [34]. The genome sequence was converted to colour space using bowtie-build and this is used as a reference for alignment of ChIP-seq reads by Bowtie [85]. Using SAMtools [86] aligned sam files were converted to bam files and used for further analysis. Correlation of bam files, peak profiles, heatmaps and cluster analysis were carried out by using deepTools [87]. Identification of ChIP-seq peaks, differential enrichment of peaks and annotation of these peaks were carried out by HOMER [88]. Motif enrichment analysis was performed by extracting 500 bp regions flanking the H3K27ac peak center and subjected to MEME-ChIP using MEME-Suite [35]. Following MEME-ChIP, the obtained motifs were submitted to TOMTOM in MEME-Suite to identify the TF that binds to the intergenic region. RNA-seq data generated previously in the lab [19] was used to show transcription over the selected peak regions (SAMN08966965).

### Identification of differentially expressed genes

Data from NCBI-SRA was downloaded and used for this analysis. Here, sequence reads were aligned to the reference genome using HISAT2 [89]. Aligned bam files were used to get count matrix files using featureCounts function of Subread package [90]. Differential gene expression analysis in head vs foot was carried out by using EdgeR [91].

### Luciferase assay

The selected intergenic regions were cloned into pGL3-Basic vector downstream of the luciferase ORF to eliminate chances of promoter-based activity. HEK293T cells were transfected with the target clones along with pRL-TK vector expressing *Renilla* luciferase as a transfection control. The cells were harvested 48 h post transfection and processed as per the given procedure using the Dual-Luciferase^®^ Reporter Assay System (Promega). The luminescence was recorded for the target firefly luciferase and *Renilla* luciferase using EnSight™ Multimode Microplate Reader (PerkinElmer). The firefly luminescence values were normalised to the *Renilla* luminescence values and the fold change was calculated over the pGL3 basic empty vector normalised values.

### Whole mount in situ hybridization

Digoxigenin-labeled RNA probes for *Hv_Wnt3a* and *Hv_Bra1* were prepared by in vitro transcriptions from templates amplified from a recombinant pCR BluntII TOPO (Invitrogen) plasmids containing the *Hv_Wnt3a* and *Hv_Bra1* cDNA using PCR. (DIG Labeling Mix—Sigma—1277073910; SP6 RNA Polymerase—Sigma—10810274001; T7 RNA Polymerase—Sigma—10881767001). Whole-mount in situ hybridization was performed on the polyps as described [92] with the following changes. Treatment with proteinase-K was performed for 5 min and heat-inactivation of the endogenous alkaline phosphatases was done at 70 °C for 15 min in 1X SSC. Digoxigenin-labeled RNA probe at a concentration of 150 ng/ml was used for hybridization at 59 °C. The post-hybridization washes were performed using 1X SSC-HS gradients. After staining with 50% NTMT/50% BM-purple AP substrate for 1 h at room temperature, the animals were mounted in 80% glycerol for imaging. Imaging was performed using Olympus MVX10 stereomicroscope.

## Supplementary information

**Additional file 1.** Additional figures.

**Additional file 2: Table S1.** Bed file for locations of ChIP-Seq and RNA-Seq peaks for genic regions in *Hydra*.

**Additional file 3: Table S2. **(**a**) ChIP-Seq peak annotation for H3K4me3 in whole Hydra polyps. (**b**) ChIP-Seq peak annotation for H3K27ac in whole Hydra polyps. (**c**) ChIP-Seq peak annotation for H3K9ac in whole Hydra polyps.

**Additional file 4: Table S3.** BED file for H3K27ac ChIP-Seq peaks on intergenic regions.

**Additional file 5: Table S4.** BED file for H3K27ac ChIP-Seq peaks on intergenic regions enriched after ALP treatment.

**Additional file 6: Table S5.** (**a**) Annotated protein list for genes nearest to intergenic regions with H3K27ac enriched after ALP treatment in cluster1. (**b**) Annotated protein list for genes nearest to intergenic regions with H3K27ac enriched after ALP treatment in cluster2. (**c**) Annotated protein list for genes nearest to intergenic regions with H3K27ac enriched after ALP treatment in cluster3. (**d**) Annotated protein list for genes nearest to intergenic regions with H3K27ac enriched after ALP treatment in cluster4.

**Additional file 7: Table S6. **(**a**) Gene lists for the four clusters in Fig 3c. (**b**) List of genes upregulated in the head. (**c**) List of genes downregulated in the head.

**Additional file 8: Table S7.** Enrichment of PFAM terms in network analysis. Clustering of the genes identified in the STRING db and colour codes for the clusters generated.

**Additional file 9: Table S8.** (**a**) Primer used for ChIP-qRT-PCR. (**b**) Relative luminiscence readings for the luciferase assay performed with the target intergenic regions (Source data for Fig. 4b) (**c**) ddCt values of H3K27ac occupancy estimated using ChIP-RT qPCR (Source data for Fig. 4c) (**d**) ddCt values of H3K27ac occupancy estimated using ChIP-RT qPCR (Source data for Fig. 5d).

## Data Availability

The results of MEME-Suite motif analysis related data are available in Figshare at the https://doi.org/10.6084/m9.figshare.7993853. The ChIP-Seq data is available in NCBI under the following IDs: SRR8893489-97. For the differential gene expression analysis of head versus food transcriptomes, the data was used from the following IDs: (SRR6815024-29). The source data for Fig. 4b, c and 5d is available in Additional file 9: Table S8.
