# Supplementary material for: Epigenomic landscape of enhancer elements during Hydra head organizer formation
Source: Epigenetics Chromatin. 2020 Oct 12;13:43. doi: 10.1186/s13072-020-00364-6 (PMC7552563; doi:10.1186/s13072-020-00364-6)
Supplement: Supplementary file 1 — Additional file 1. Additional figures. [file 13072_2020_364_MOESM1_ESM.pdf]

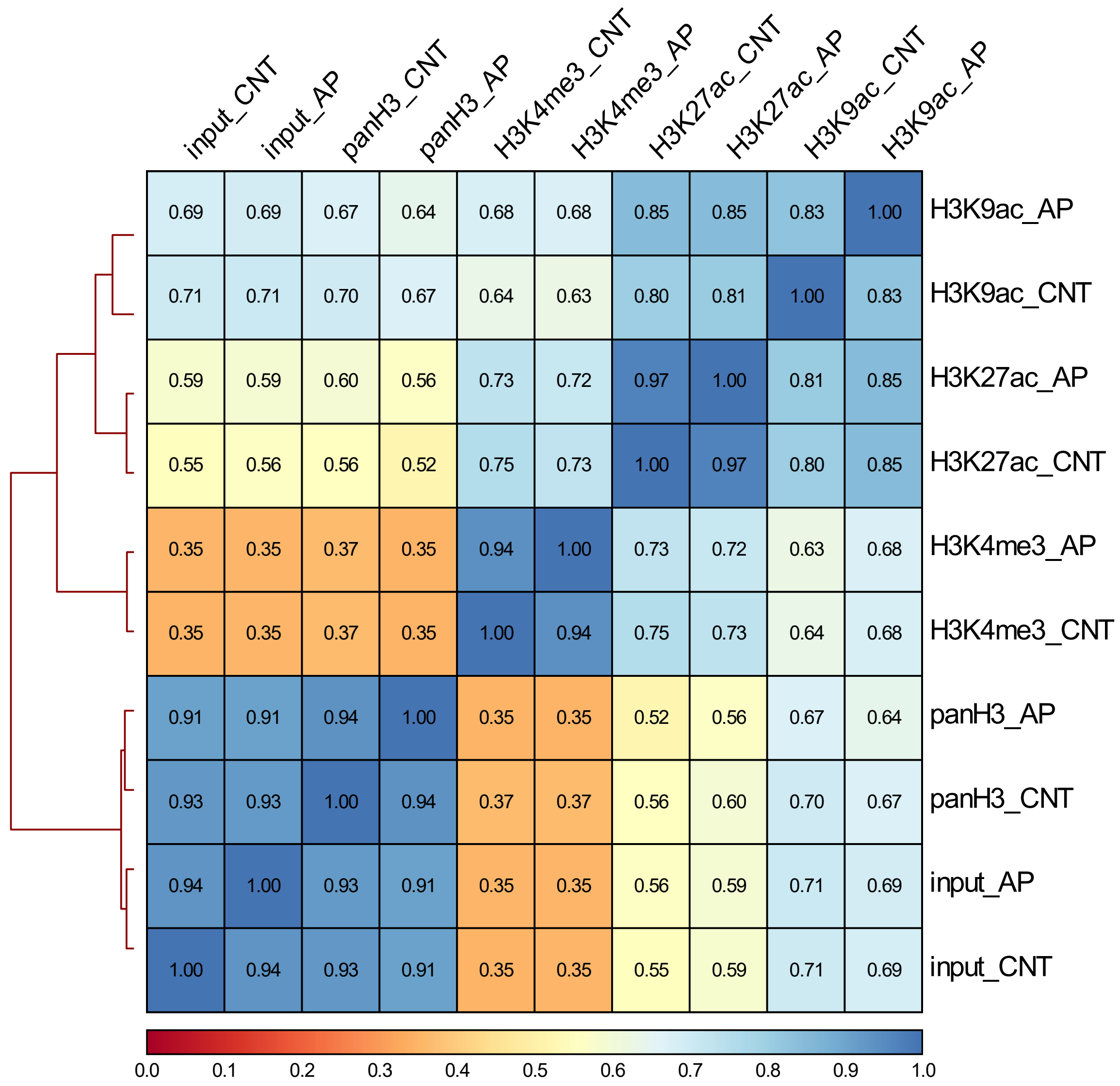

Figure S1: Correlation of the reads obtained for all the histone modifications in the ChIP sequencing data. This correlation plot shows a greater correlation between the acetylation marks, H3K27ac and H3K9ac than between the H3K4me3 methyl-mark and the two acetylation marks. There is also good correlation for each mark regarding its occupancy in control and ALP treatments. This represents the *in situ* scenarios for these histone modifcations at various locations on the chromatin (Fig. 1A).

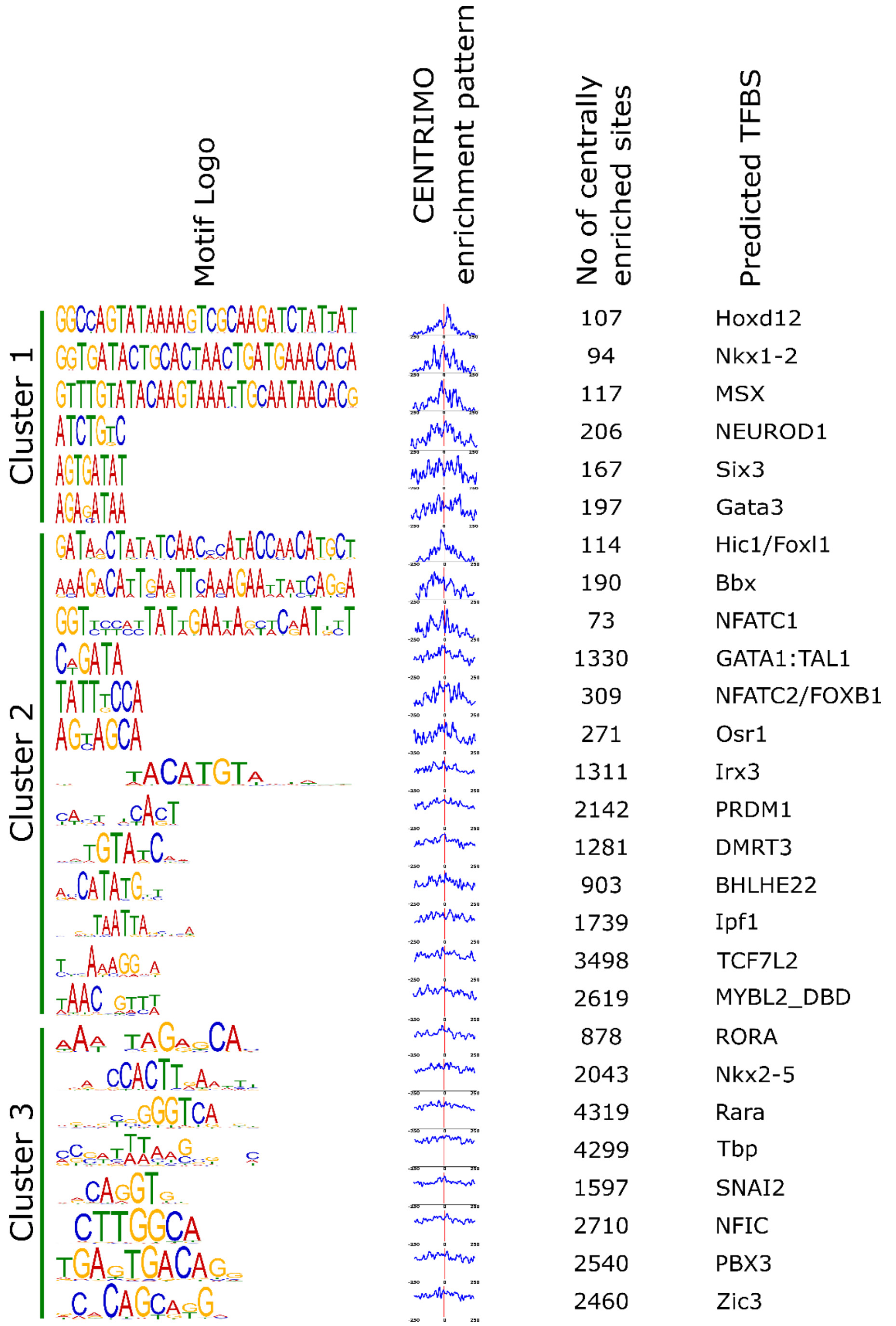

Figure S2: Central enrichment of motifs on the intergenic regions displaying occupancy of the H3K27ac, H3K9ac and H3K4me3 modifications. Upon identifying the intergenic regions displaying occupancy of H3K27ac mark, they were retrieved. These were classified into 3 k-means clusters based on combinatorial occupancy of the three activating histone marks and subjected to motif enrichment analysis using the MEME suite package (Fig. 2A). The first column shows the motif identified on the intergenic regions using the DREME and FIMO tools in the online MEME suite package. The 2nd column shows the pattern of motif enrichment on the DNA sequences and the 3rd column shows the number of centrally enriched peaks for each of the transcription factors listed in the 4th column. The total number of motifs identified for each transcription factor in each cluster is given in Fig. 2E.

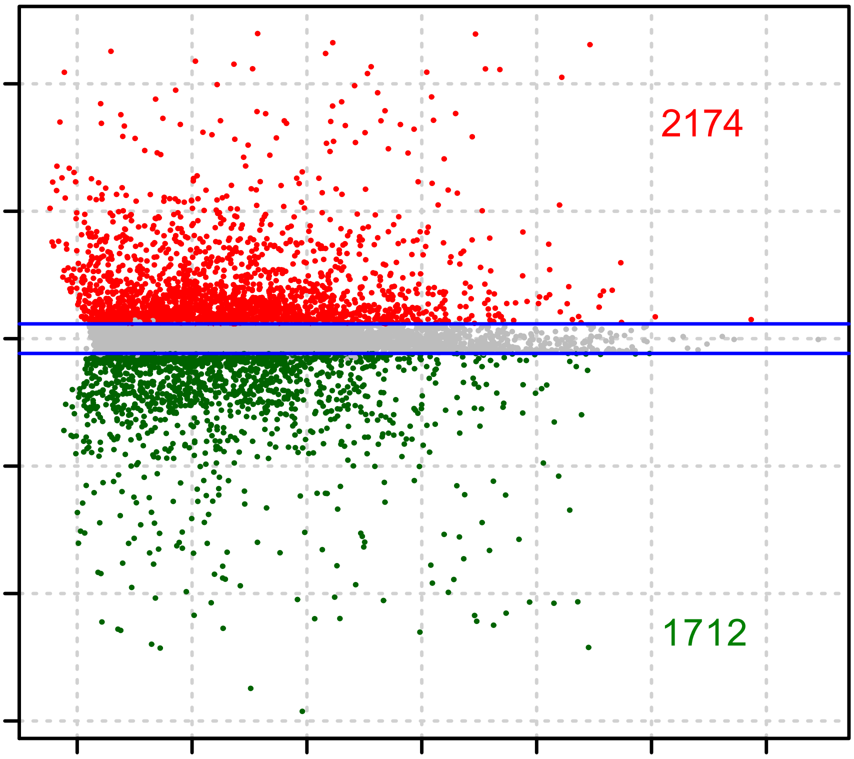

Figure S3: Differentially expressed transcripts in head vs foot. To identify genes having head-specific expression in *Hydra*, a publicly available dataset was analysed (IDs: SRR6815024-29). The smear plot represents the differentially regulated transcripts in head specific regions with the red dots representing the genes upregulated in a head specific manner and the green dots depict the downregulated genes in head. The head specific genes were used for identifying genes neighbouring to intergenic regions that are differentially modified in response to Wnt/β-catenin signalling as seen in Fig. 3C, 3D and 3E.

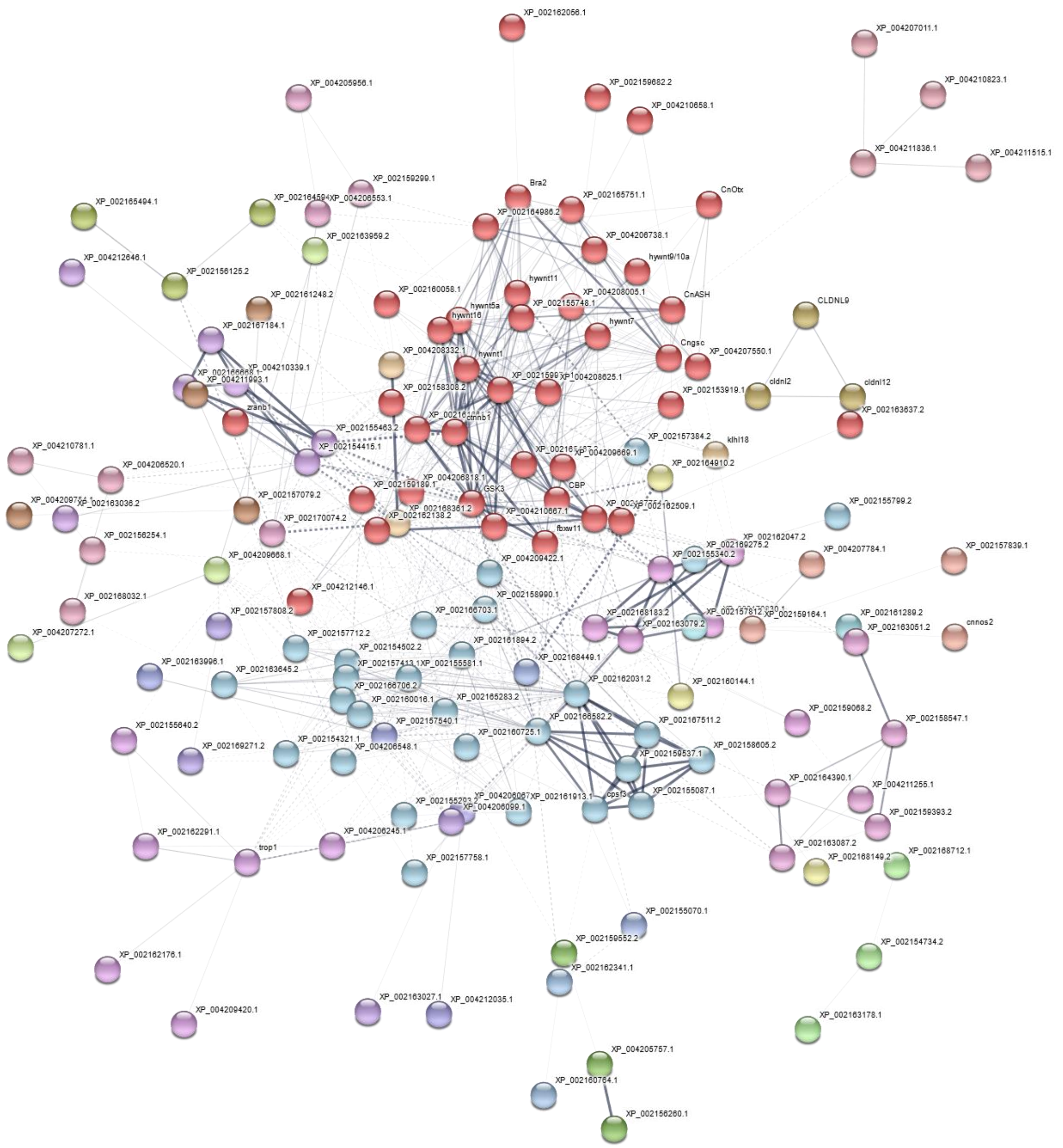

Figure S4: Networks obtained after enrichment analysis and clustering using STRINGdb. Upon identification of a set of head-related genes neighbouring to the intergenic regions that respond to activated Wnt/β-catenin signalling, they were subjected to a network and interactome analysis. Interaction network obtained using STRING database for the genes commonly found in head and cluster3_4 of Fig. 3C. The KEGG component enrichment is shown in Supplementary Table 7a and the colour codes for the clusters are given in Supplementary Table 7b.

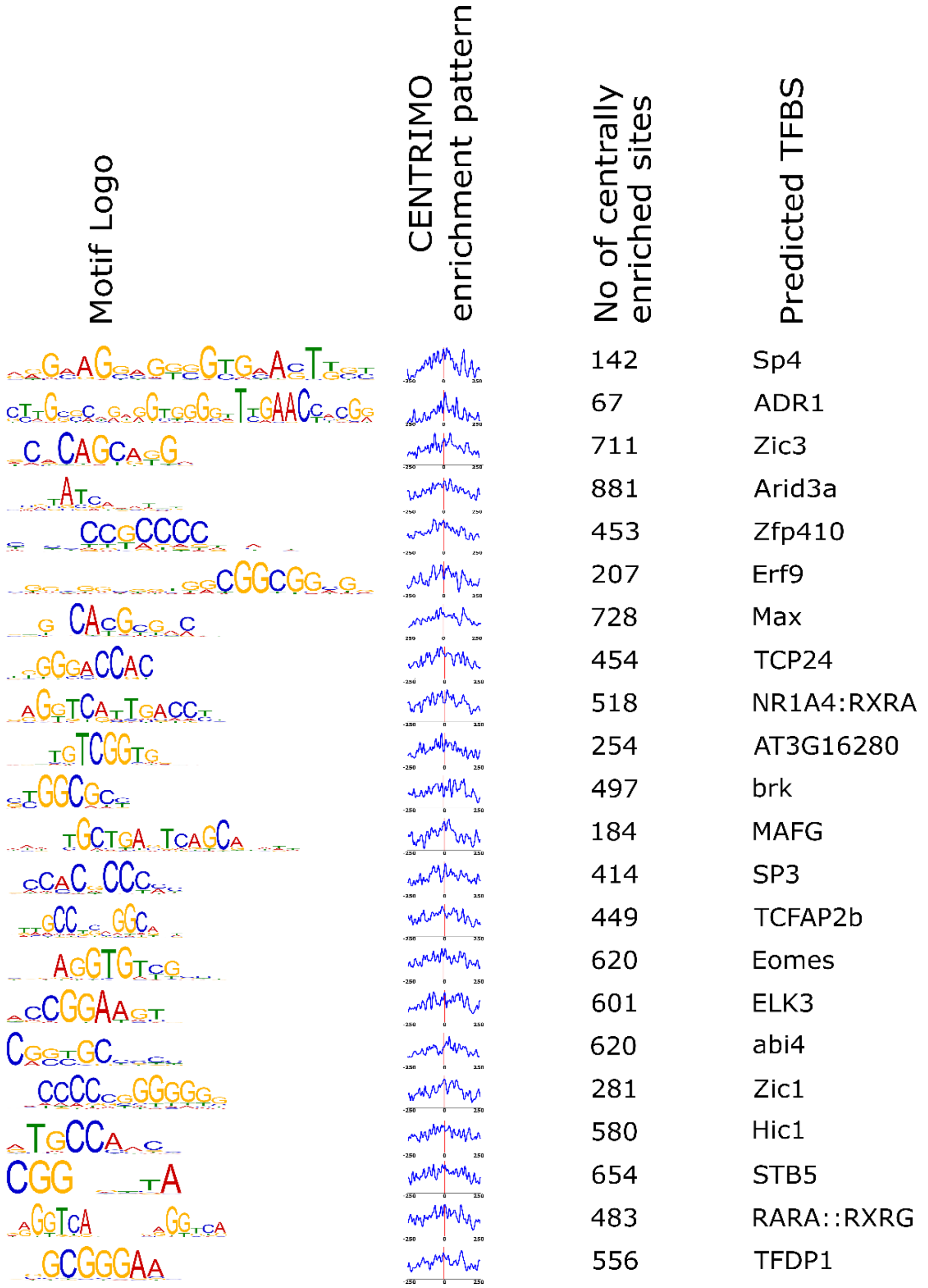

Figure S5: Central enrichment of motifs on the intergenic regions displaying higher occupancy of the H3K27ac neighbouring to genes having expression in head. Intergenic regions showing enhanced H3K27ac occupancy and neighbouring to the genes which display expression in the *Hydra* head were subjected to motif enrichment analysis in the MEME suite (Fig. 3C, 3D). The first column shows the motif identified on the intergenic regions using the DREME and FIMO tools in the online MEME suite package. The 2nd column shows the pattern of motif enrichment on the DNA sequences and the 3rd column shows the number of centrally enriched peaks for each of the transcription factors listed in the 4th column. The total number of motifs identified for each transcription factor is given in Fig. 3E.

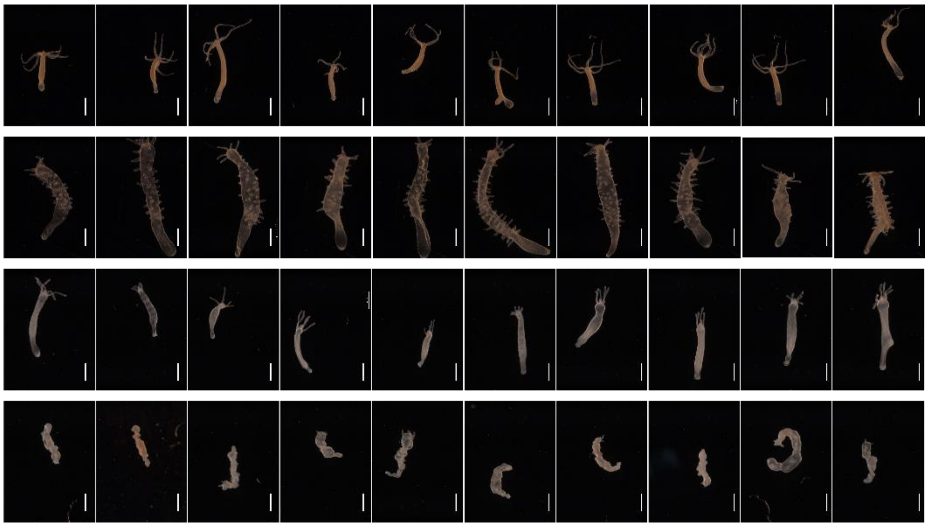

Figure S6: Morphology of *Hydra* polyps upon treatment with pharmacological inhibitors. Family images of polyps treated with Vehicle (DMSO), 5 µM Alsterpaullone (ALP), 3 µM GSK-J4 which inhibits the JMJD3/UTX histone lysine demethylases and a combinatorial treatment of 3 µM GSK-J4 and 5 µM ALP. As seen in Fig. 5A, B, treatment with ALP activates the Wnt/β-catenin signalling pathway leading to formation of ectopic tentacles all over the *Hydra* polyp. GSK-J4 is an inhibitor of KDM6A/6B histone demethylases and application of this inhibitor in combination with ALP leads to a rescue in the ectopic tentacle phenotype. (Scale bar = 500 µm).

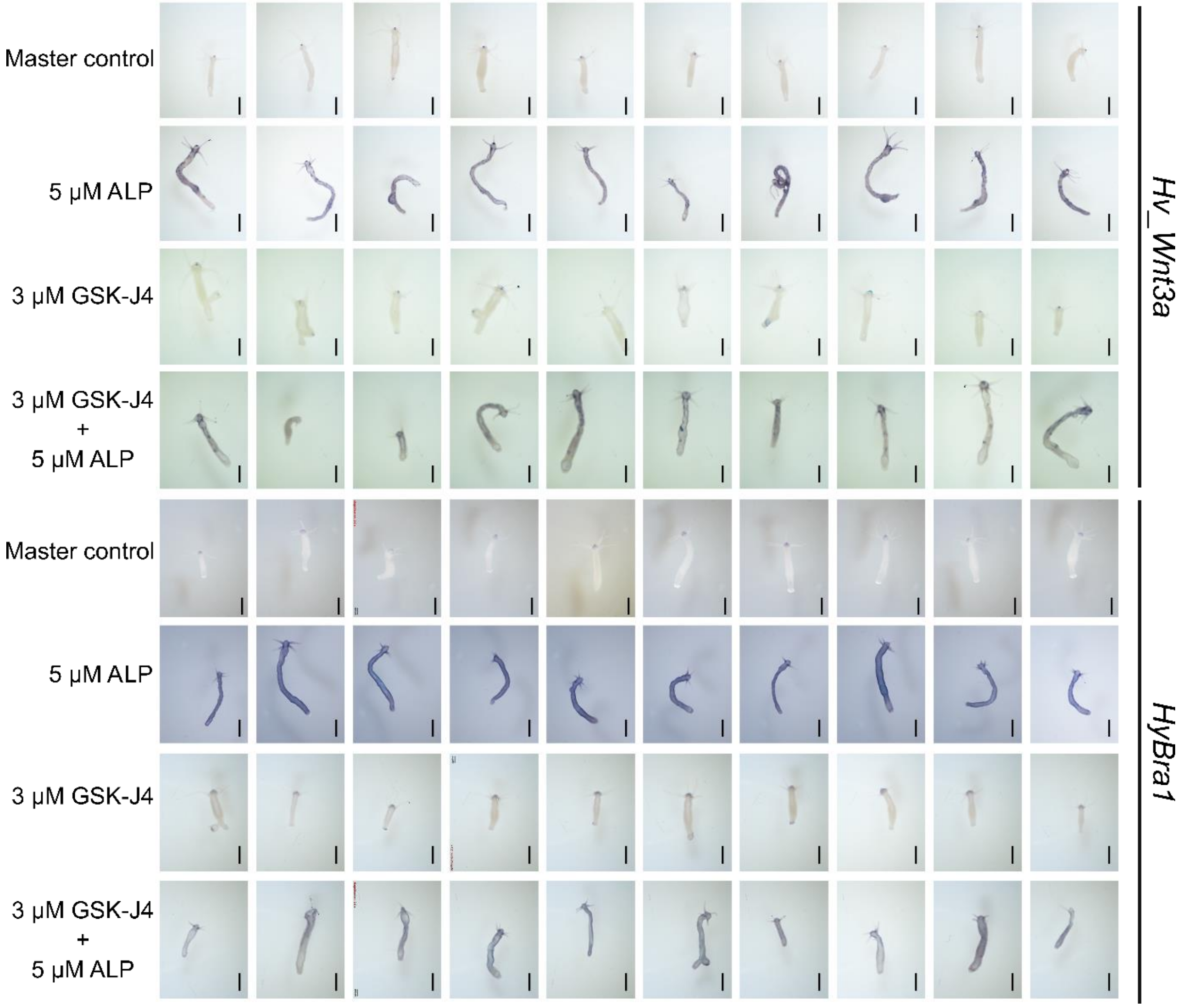

Figure S7: Whole mount *in situ* hybridisation of polyps treated with pharmacological inhibitors. Family images of whole mount *in situ* hybridisation for *Hv_Wnt3a* and *HyBra1* on *Hydra* polyps treated with Vehicle (DMSO), 5 µM ALP, 3 µM GSK-J4 which inhibits the JMJD3/UTX histone lysine demethylases and a combinatorial treatment of 3 µM GSK-J4 and 5 µM ALP. As seen in Fig. 5C, treatment with ALP activates the Wnt/β-catenin signalling pathway leading to formation of multiple head organizer centres expressing *Hv_Wnt3a* and *HyBra1* all over the body column of *Hydra*. This changes the patterning of the *Hydra* body axis. GSK-J4 is an inhibitor of KDM6A/6B histone demethylases and application of this inhibitor in combination with ALP leads to a reduction of regions expressing genes that are a signature of the *Hydra* hypostome. (Scale bar = 500 µm).
